# Supplementary material for: Vericiguat suppresses ventricular tachyarrhythmias inducibility in a rabbit myocardial infarction model
Source: PLoS One. 2024 Apr 16;19(4):e0301970. doi: 10.1371/journal.pone.0301970 (PMC11020759; doi:10.1371/journal.pone.0301970)
Supplement: S1 Fig — The MI region is indeed the area with scar tissue. We could visually define the scar tissue (as shown by the white scar in the left upper panel), identified it with a fluorescent dye (as indicated by the green area in the left lower panel, as healthy tissue emits orange fluorescence), and confirmed it on the optical mapping images (MI area displayed only obscure signals, as shown by the signals noise in the right panel). Since the signals in the MI zone are faint, we did not include the MI in the data analyses. (PDF) [file pone.0301970.s001.pdf]

S1 Fig

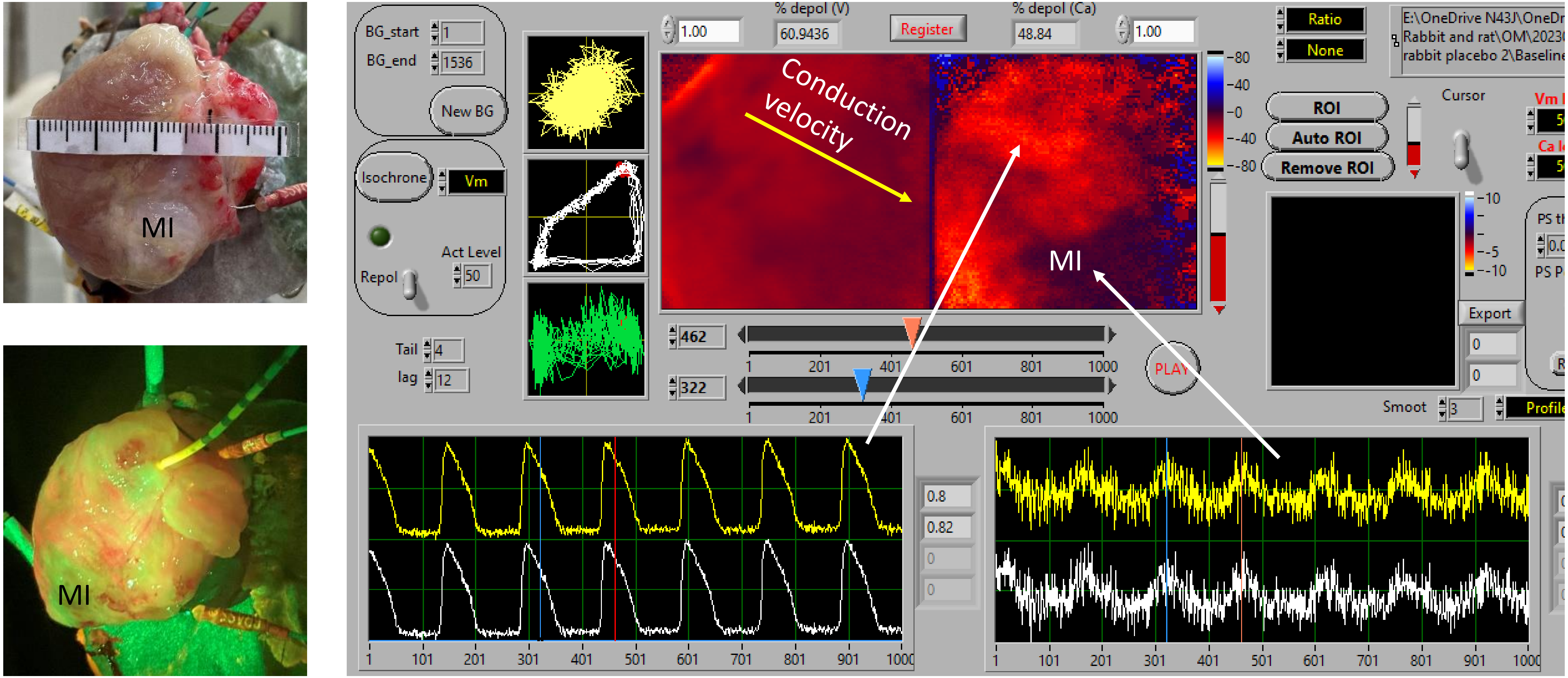
MI
MI
BG_start
1
BG_end
1536
New BG
Isochrone
Vm
Act Level
50
Repol
Tail
4
lag
12
1.00
% depol (V)
60.9436
Register
% depol (Ca)
48.84
1.00
Conduction velocity
MI
-80
-40
-0
-40
-80
Ratio
None
ROI
Auto ROI
Remove ROI
Cursor
462
322
PLAY
Export
Smoot
3
0.8
0.82
0
0
1
101
201
301
401
501
601
701
801
901

- **S1 Figure**. Visually and optical mapping images to identify the scars. The MI region is indeed the area with scar tissue. We could visually define the scar tissue (as shown by the white scar in the left upper panel), identified it with a fluorescent dye (as indicated by the green area in the left lower panel, as healthy tissue emits orange fluorescence), and confirmed it on the optical mapping images (MI area displayed only obscure signals, as shown by the signals noise in the right panel). Since the signals in the MI zone are faint, we did not include the MI in the data analyses.
