# Supplementary material for: Vericiguat suppresses ventricular tachyarrhythmias inducibility in a rabbit myocardial infarction model
Source: PLoS One. 2024 Apr 16;19(4):e0301970. doi: 10.1371/journal.pone.0301970 (PMC11020759; doi:10.1371/journal.pone.0301970)
Supplement: S2 Fig — A. VERP and the representative ECG traces. B and C. Action potential duration (APD) at pacing cycle length 300 ms and 200 ms, respectively, and the representative AP traces. D and E. CaD at pacing cycle length 300 ms and 200 ms, respectively, and the representative Cai traces. The comparisons of the representative traces were acquired from the same rabbit. APD80, action potential duration measured to 80% repolarization; CaD80, intracellular calcium transient duration measured to 80% repolarization; VERP, ventricular effective refractory period. (PDF) [file pone.0301970.s002.pdf]

S2 Fig

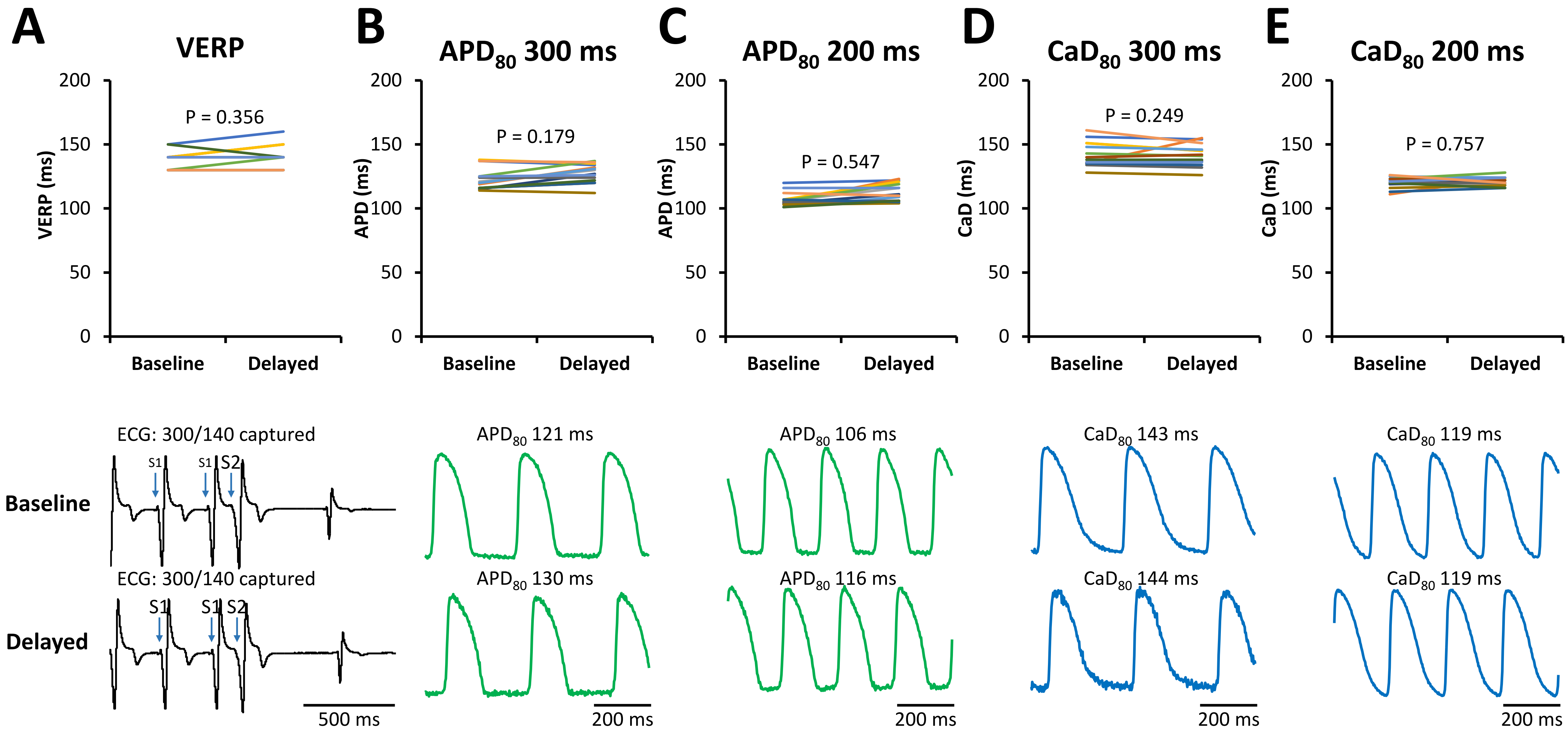
A
VERP
P = 0.356
VERP (ms)
200
150
100
50
0
Baseline
Delayed
B
APD80 300 ms
P = 0.179
APD (ms)
C
APD80 200 ms
P = 0.547
D
CaD80 300 ms
P = 0.249
CaD (ms)
E
CaD80 200 ms
P = 0.757
ECG: 300/140 captured
S1
S2
Baseline
Delayed
500 ms
APD80 121 ms
APD80 130 ms
APD80 106 ms
APD80 116 ms
CaD80 143 ms
CaD80 144 ms
CaD80 119 ms
CaD80 119 ms
200 ms

- **S2 Fig.** VERP, APD_{80} and CaD_{80} at baseline and the delayed phase (N = 7). **A.** VERP and the representative ECG traces. **B and C.** Action potential duration (APD) at pacing cycle length 300 ms and 200 ms, respectively, and the representative AP traces. **D and E.** CaD at pacing cycle length 300 ms and 200 ms, respectively, and the representative Ca_i traces. The comparisons of the representative traces were acquired from the same rabbit. APD_{80}, action potential duration measured to 80% repolarization; CaD_{80}, intracellular calcium transient duration measured to 80% repolarization; VERP, ventricular effective refractory period.
