# Supplementary material for: Vericiguat suppresses ventricular tachyarrhythmias inducibility in a rabbit myocardial infarction model
Source: PLoS One. 2024 Apr 16;19(4):e0301970. doi: 10.1371/journal.pone.0301970 (PMC11020759; doi:10.1371/journal.pone.0301970)
Supplement: S3 Fig — A. Summarized results of the cardiac alternans at baseline and the delayed phase. B. Representative Vm and Cai traces. The comparisons of the representative traces and maps were acquired from the same rabbit. PCL, pacing cycle length. (PDF) [file pone.0301970.s003.pdf]

S3 Fig

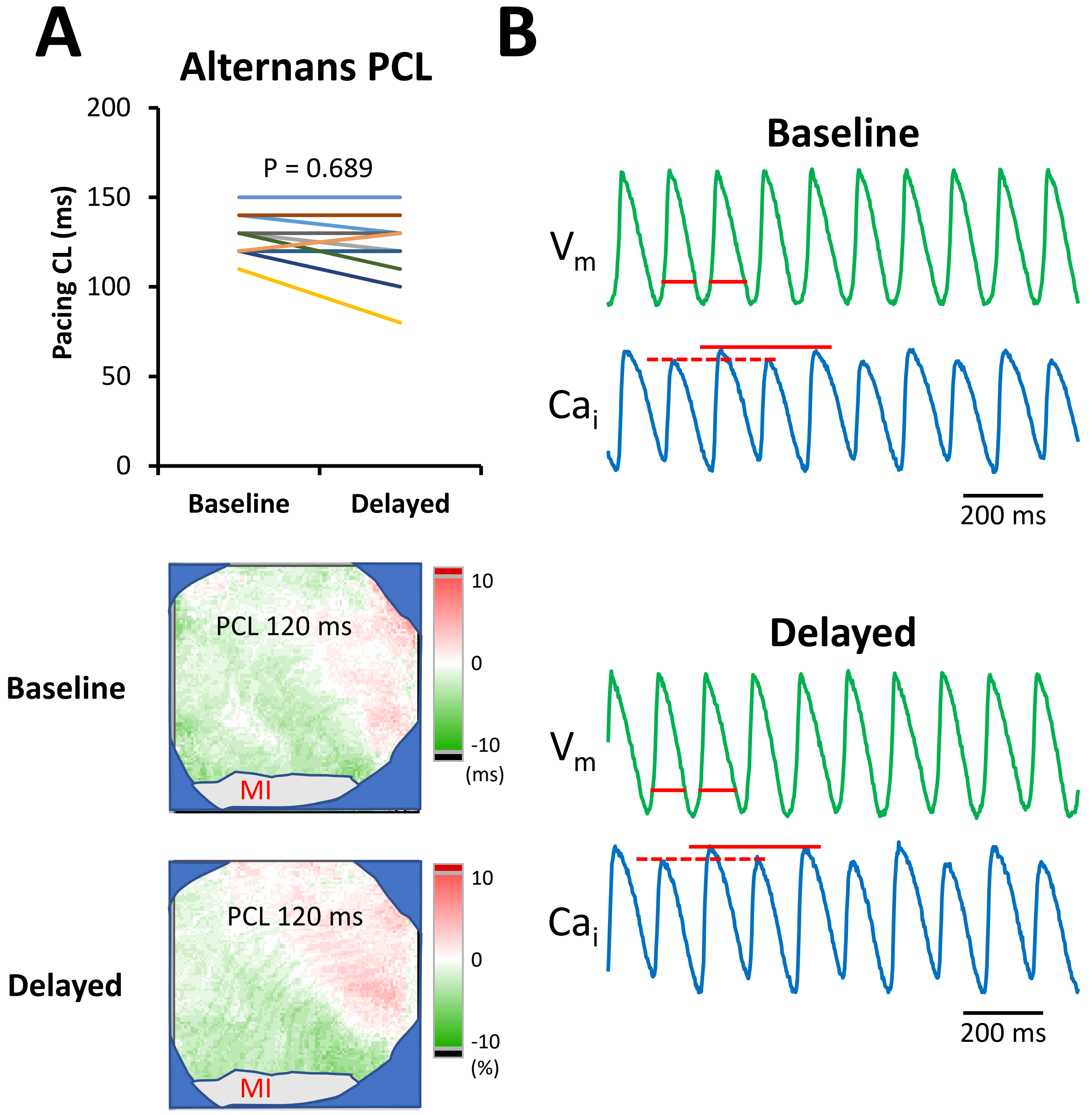
A
Alternans PCL
200
150
100
50
0
Pacing CL (ms)
P = 0.689
Baseline
Delayed
Baseline
PCL 120 ms
MI
10
0
-10
(ms)
Delayed
PCL 120 ms
MI
10
0
-10
(%)
B
Baseline
Vm
Cai
200 ms
Delayed
Vm
Cai
200 ms

- **S3 Fig.** Alernans maps at baseline and the delayed phase (N = 7). **A.** Summarized results of the cardiac alternans at baseline and the delayed phase. **B.** Representative V_m and Ca_i traces. The comparisons of the representative traces and maps were acquired from the same rabbit. PCL, pacing cycle length.
