# Supplementary material for: Vericiguat suppresses ventricular tachyarrhythmias inducibility in a rabbit myocardial infarction model
Source: PLoS One. 2024 Apr 16;19(4):e0301970. doi: 10.1371/journal.pone.0301970 (PMC11020759; doi:10.1371/journal.pone.0301970)
Supplement: Attachment — Submitted filename: Response to reviwers comments 2.pdf [file pone.0301970.s005.pdf]

<u>Responses to Reviewers' Comments</u>

Reviewer #2: In their study "Vericiguat suppresses ventricular tachyarrhythmias inducibility in a rabbit myocardial infarction model" Chou et al. investigate the effect of a vericiguat, a stimulator of guanylate cyclase, onto the susceptibility of infarcted isolated rabbit hearts to ventricular arrhythmias using optical mapping. The study is examplary for demonstrating the utility of optical mapping for studying the mechanisms underlying the arrhythmogenesis of compounds and disease in electrophysiological studies. The study is scientifically sound, rigorously executed and includes a detailed discussion putting their findings into perspective. I have only minor concerns regarding some of the methodological approaches and presentation of the results:

Response: We appreciate the reviewer's insightful comments after the detailed review. They have greatly helped us improve the manuscript.

1. Please use a format with figures and figure captions in place with figure captions next to the figure and the figure close to where referenced in the text. That way I do not have to scroll back and forth from the beginning to the end of the document all the time.

Response: We appreciate the reviewer for the comment and made an additional version to make the reviewing process more comfortable.

2. The study lacks a conflict of interest statement.

Response: We appreciate the reviewer for the comment and have add conflict of interest statement accordingly (both ICMJE Disclosure Form and a statement in the manuscript).

3. Please add age and sex of rabbits.

Response: We appreciate the reviewer's advice. The average age of rabbits during the optical mapping study was 25.1 ± 2.5 weeks. For this study, we included a total of 7 female and 7 male rabbits in the optical mapping analyses. Accordingly, we have revised the Results sections (lines 168-171).

4. Are there representative APD maps that complement the bar plot in Fig. 2A?

Response: We appreciate the reviewer's suggestion and have incorporated APD and Cai duration maps into Figure 2.

5. Please add the number of hearts in Fig. 2 A-E.

Response: We have added heart number to Figure 2 accordingly.

6. The bar plots do not reveal the number of individual measurements and their spread. Please use a scatter or stripchart plot in addition to bar plots, e.g. as shown here: https://www.ashander.info/notes/barchart-alternatives-in-base-R_files/figure-html/scatter-v-factors-1.png

Response: We appreciate the reviewer's suggestion and have revised the bar charts using a connected scatterplot, as per the comment.

7. Please add an overlay of the traces for one AP or CA-transient so that one can see the effect more clearly or align them in time. The traces as they are presented right now are not helpful for evaluating a change in duration.

Response: We appreciate the reviewer's suggestion and have replaced the original AP and Cai traces with overlaid traces.

8. line 186: I feel like the statement "vericiguat therapy significantly slowed the ventricular myocardial conduction" is not supported by the presented data. In the bar plot in Fig. 3A,B the difference is hardly noticeable and I am surprised it is significant based on the p-value (which is a poor measure for statistical significance) given the large error bars. See 2 points below.

Response: We appreciate the reviewer's comment. Due to the variable conduction velocity among different hearts, the variation was significant. Nevertheless, the paired comparisons still demonstrated statistically significant differences between the baseline and vericiguat therapy. We have replaced the original bar graphs with connected scatterplots.

9. Fig. 3. I do not understand how CV was measured. Please add points from where to where and along which path the distance and CV was measured in the figure.

Response: We appreciate the reviewer's comment. In response, we added points to illustrate the path and the locations where conduction velocity (CV) was measured. Taking Figure 3A baseline as an example, the pacing site is located on the left (indicated by the pacing site symbol), and the arrow indicates the conduction path. We measured the distance between the arrow tail and the arrowhead (1.56 cm in this heart) and the conduction duration (22 ms). The calculated conduction velocity was 70.80 cm/s.

10. Given that the change in CV in the bar plots is not very large: Could this measurement be improved? For instance, the authors could measure the distance between isochrones for every point on the isochrone and compute a CV map and average over the data. Maybe this would produce a more distinct separation between baseline and Vericiquat.

Response: We appreciate the reviewer's comment. The issue is not a measurement problem. Due to the variable conduction velocity among different hearts, the variation was significant.

11.Fig. 3: Why are there Ca alternates but no V alternates?

Response: We thank the reviewer for raising the question. Membrane potential (V_m) alternans manifested as changes in action potential duration (APD) from short to long, while calcium (Ca_i) alternans presented as amplitude changes from low to high. We observed both V_m and Ca_i alternans.

12.lines 195-198 and Fig. 4B: I would like to see more representative traces that highlight the shortening of the tau value with overlays of the baseline and vericiguat traces.

Response: We appreciate the reviewer's comment. Because the Cai decay depends on the Cai curve and its fitting. Additional overlaid traces of the baseline and vericiguat might not be good to show the changes (for example the below traces).

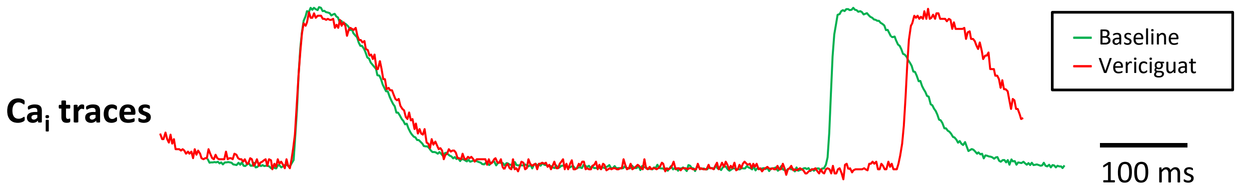

13.Please provide videos for optical mapping data.

Response: We appreciate the reviewer's comment. We included the representative videos in the supplementary data.

Please refer to

https://1drv.ms/f/s!AnJU7ssgUKgtg8FXi8YU7EWNbhZ1Fw?e=SQeDSm

14.Please provide the video raw data in an appropriate format (e.g. a Matlab or Numpy .mat / .npy file which includes an array) for Fig. 5 D, G.

Response: The raw data for MiCAM images is in "rsd" format. Unfortunately, we were unable to transform the format into either "mat" or "npy." We have uploaded the raw data in "rsd" format, zipped into a file ("Pacing induced VT raw data for Fig 5.zip").

Please refer to

https://1drv.ms/f/s!AnJU7ssgUKgtg8FXi8YU7EWNbhZ1Fw?e=SQeDSm

15.line 233: how did the shortening of tau / acceleration of Ca homeostasis contribute to suppression of alternans?

Response: We appreciate the reviewer's question. The efficiency of Cai removal, or cytosolic Ca2+ sequestration, is associated with the development of Ca2+-driven alternans. Cai removal is mostly dependent on the activity of SERCA and the activity of the NCX. Low efficiency of cytosolic Ca2+ sequestration leads to the development of Ca2+-driven alternans. Therefore, the overall effects of sGC stimulation on Ca2+ include enhanced Ca2+ reuptake by SERCA and increased Ca2+ efflux/influx by NCX, leading to accelerated Cai homeostasis, a shorter Cai decay, and suppression of Cai alternans. We have added some descriptions in the Discussions section.

16.line 58: missing reference

Response: We thank the reviewer for the reminder.

17.line 48: coronary artery disease (CAD) contributes

Response: We thank the reviewer for the reminder. We have revised the text accordingly.

18.lines 318: Why did the authors choose this much higher concentration and what happens when the dosage is reduced, let’s say to 1 micro mol/L?

Response: According to previous publications (J Med Chem 60, 5146-5161; Am J Health Syst Pharm 78, 1021-1023), the EC50 of vericiguat for sGC activation is 1.005 ± 0.145 mmol/L. As reported by Cai et al. (Ann Transl Med. 2022; 10: 662.), vericiguat at a dose of 1 to 10 μmol/L is considered safe and capable of promoting an increase in cGMP in cardiomyocytes. Therefore, we chose a larger dose to ensure the pharmacological effects in the animal model.

19.line 323: missing reference

Response: We thank the reviewer for the reminder.

20.In lines 298-299 the authors mention electrophysiological changes in ischemic failing hearts including slowing of CV and prolongation of AP, which they also report with vericiguat. How can these changes be pro- and anti-arrhythmic at the same time?

Response: We appreciate the reviewer’s comment. Many anti-arrhythmic agents exhibit both anti- and pro-arrhythmic properties simultaneously. The overall composite effects determine whether an agent acts as an anti- or pro-arrhythmic agent for a specific arrhythmia (e.g., ventricular tachycardia) in a particular context (e.g., ischemic heart). In this MI model, we believe the composite effects of vericiguat contribute to its anti-arrhythmic properties. These reasons include the enhancement of conduction, suppression of cardiac alternans, and inhibition of arrhythmic triggers. The collective effects render vericiguat anti-arrhythmic.

On the other hand, prolongation of APD increases the wavelength (wavelength = APD × CV), leading to a higher probability of wave break (Weiss JN, et al. Circulation. 2005; 112: 1232-1240). In this study, the decrease in CV

after vericiguat therapy might offset the pro-arrhythmic effects of APD prolongation regarding the wavelength, preventing the formation of wave breaks during rapid pacing. The decrease of CV might protect the rabbit hearts from wave breaks and the development of ventricular tachyarrhythmias.

We have made revision in the Discussions section (lines 297-301).
