# Supplementary material for: Vericiguat suppresses ventricular tachyarrhythmias inducibility in a rabbit myocardial infarction model
Source: PLoS One. 2024 Apr 16;19(4):e0301970. doi: 10.1371/journal.pone.0301970 (PMC11020759; doi:10.1371/journal.pone.0301970)
Supplement: Attachment — Submitted filename: Responses to reviewers comments.docx [file pone.0301970.s006.docx]

**Responses to reviewers’ comments**

**Reviewer #1:** All comments have been addressed which i thank the Authors for. Please check grammar and spelling throughout the manuscript though- for instance on line 272, cGC is stated instead of sGC.

**Response**: We appreciate the reviewer’s comments and effort. We have revised the manuscript accordingly.

**Reviewer #2**: Thank you very much for the detailed response and revision of the manuscript. I have a few remaining minor concerns, the main concern being that the measured effects (APD / CV) are very small and I would like to verify the effect in the raw data myself (PlosONE specifically asks me whether all raw data was made available).

**Response**: We appreciate the reviewer’s comments and effort. We have uploaded the videos, as well as the optical mapping raw data, as suggested.

- Please mark all revised parts of your manuscript in blue, otherwise it is difficult to confirm which parts were revised.

**Response**: Thank you for the suggestion. Previously, we uploaded a file with yellow-highlighted texts to indicate the revisions made in the R1 version. In this R2 revision, we have marked the revised texts in blue (including the revised texts in R1).

- Thank you very much for providing raw data (Pacing induced VT raw data for Fig 5). Could you please provide raw data for Figs. 2 and 3 which provides a comparison of baseline and Vericiguat? Please provide several paired corresponding videos with baseline and Vericiguat so that I can reproduce and verify the plots in Figs. 2 and 3.

**Response**: We appreciate the reviewer’s comments. In the R2 revision, we have uploaded several corresponding videos and raw data at baseline and with vericiguat therapy. Per the request from editorial office, we have removed the figures and legends from the main text. We apologize for the inconvenience.

Please refer to

Videos:

[Videos for Fig 2 and 3](https://1drv.ms/f/s!AnJU7ssgUKgtg8QdlGG-95PAVomUKw?e=to10Pn)

<https://1drv.ms/f/s!AnJU7ssgUKgtg8QdlGG-95PAVomUKw?e=to10Pn>

Optical mapping raw data:

[OM raw data](https://1drv.ms/f/s!AnJU7ssgUKgtg8Ia4nMt8oC9wxZGOw?e=DjEexX)

<https://1drv.ms/f/s!AnJU7ssgUKgtg8Ia4nMt8oC9wxZGOw?e=DjEexX>

- The authors should phrase the significance of the measured APD and CV changes more carefully. They may be statistically significant, but still small.

**Response**: We appreciate the reviewer’s suggestions. We recognize the small changes in APD and CV. However, the significant small CV reduction and APD prolongation might contribute, at least partly, to the observed electrophysiological effects of vericiguat in the chronic MI model. Although the APD and CV changes were small, the combination of the changes as well as Ca homeostasis led to suppression of discordant alternans and functional conduction block/ wave break. Thereby, the ventricular arrhythmias inducibility was also suppressed. We have revised the text to mention the small changes in APD and CV in the Discussions (line 236; lines 364-370).

- The rabbits are very young (6 months). The limitations section should list this as a potential limitation (would the results change with older rabbits?).

**Response**: Certainly, the rabbits are relatively young. We appreciate the reviewer for the comment and concern. We have revised the limitations (lines 371-372).
